# Kirkes' Hand-Book of Physiology

**Published:** 1899-12

**Authors:** 


**Kirkes' Hand-Book of Physiology.** By W. D. HALLIBURTON, M.D., F.R.S. Fifteenth Edition. Pp. xx., 872. London: John Murray. 1899.

Because it is especially distinguished by the fact that it treats of histology as well as physiology proper, and because the book is popular with the student, a new edition has become necessary after many short intervals, the last only a little over two years. The fourteenth edition was practically a new book, retaining the old form and scope of the previous work; the fifteenth incorporates all the important facts which have been since discovered, but with little alteration otherwise. The illustrations throughout the book are exceedingly good, and more especially those on the nervous system.

